# The Royal Devon and Exeter Hospital

**Published:** 1907-08-31

**Authors:** 


### HOSPITAL ADMINISTRATION.

### CONSTRUCTION AND ECONOMICS.

### THE ROYAL DEVON AND EXETER HOSPITAL.

The meeting of the British Medical Association at Exeter gave visitors interested in hospital management an opportunity of inspecting the local institutions, and among those which attracted most attention was undoubtedly the Royal Devon and Exeter Hospital. During the last five years important and extensive improvements have been effected in this hospital, which make it at present one of the most efficient, and, from a medical point of view, one of the best of provincial hospitals. There remain, it is true, several serious drawbacks to its complete modernisation.

To begin with, the main portion of the buildings is an old erection, not specially suited for the purposes to which it has been adapted. The hospital stands on a fine site, close to the centre of the town, yet outside the noise of the street traffic. It has its own private garden, which is unusually large and well stocked, and immediately fronting each balcony is a large, grassy quadrangle. A walk through the wards shows how actively the management has attempted to improve the conditions which exist, and the first feature that strikes the medical visitor is the thoroughness with which every ward, even the older ones, has been ventilated. Open to the air on three sides, with the windows large and permitting easy entrance of fresh air, and provided, moreover, with additional ventilating inlets, each ward is as fresh and clear as it can possibly be. It is usual on entering a hospital ward, no matter how modern or how well ventilated, for the visitor to perceive that he has passed into an atmosphere which is not exactly similar to that which pervades the other parts of the building. In provincial hospitals, where the air is purer and less fog laden, this sharp contrast is perhaps not so noticeable; in the Exeter hospital it is almost imperceptible. In the wards in the new wing the cheerful colours of the walls, the scrupulous cleanliness that pervades the whole apartment, and the large amount of sunlight that directly enters the room, combine to give an impression of brightness and comfort that would be hard to beat in any provincial or metropolitan hospital. In the wards in the old wing this cheerfulness is less apparent. There the walls retain their pristine dingy chocolate colour, not, we believe, the most exhilarating of colours for the sick patient to feed his eye upon. It would very greatly add to the attractiveness of the old wards if they were redecorated and repainted so as to bring them in line with the rooms in the new wing.

A feature of great interest in the building is the new operating theatre, the gift of a local lady, who very generously bore the whole of the expense connected with the building and furnishing of the set of rooms devoted to it. The theatre is in the old building on the top floor—an arrangement which strikes one as not having been very judiciously made, for it entails much labour in carrying patients to and from the different wards. To facilitate this translation of cases a bridge across the stairway is necessary, an arrangement which is not only clumsy and liable to break down, but which entails far more trouble than is desirable. To patients, too, this rather long journey from the wards to the theatre cannot be beneficial. It seems, however, that no other situation was possible, and under the circumstances the management has made the best of the situation.

For the theatre itself the medical visitor cannot but have the highest praise. It is roomy without being unduly large, and, owing to the excellence of the ventilation, it is the reverse of stuffy. A mistake, however, appears to have been made in providing a hot-air chamber immediately above the operating table, the skylight being a double plate-glass arrangement, with a space between in which dust accumulates, and which seems to us somewhat difficult to keep clean. With this exception—which in summer time at least may become an important one so far as the comfort of workers is concerned—there is nothing to find fault with. The furnishing of the theatre is of the newest and best pattern; the operating table, from designs specially furnished by Messrs. Down Brothers, is one of the best of its kind, and the outfit of instruments appears to provide for every possible contingency.

Attached to the theatre is the old operating room, now used for septic or unimportant cases, together with an anæsthetising room and a sterilising room. In the last mentioned is to be found the steriliser, one of the most diminutive apparatuses which we recollect ever having come across. For an institution of the size and importance of the Royal Devon and Exeter Hospital the presence of so small and manifestly inadequate a steriliser savours of the ridiculous, and the fact that dressings are sterilised and the routine sterilisation of materials for ward and theatre use is not interfered with, speaks very highly indeed for the capability of the nursing staff and those in charge of the sterilising plant—if one can dignify the apology for a really efficient steriliser by calling it such. The inadequacy of the present apparatus entails a vast amount of additional trouble and inconvenience, and there cannot be any doubt that the prompt acquisition of a more commodious plant will prove much more economical in the long run.

Attached to the theatre, and really forming part of the old operating-room, is a small apartment devoted to emergency *x*-ray work. The usefulness of the small apparatus—which, we understand, has proved thoroughly trustworthy and suitable for the purposes required—will be evident to anyone who has witnessed the laborious and circumlocutory procedure that obtains in most hospitals when cases require to be skiagraphed before operation.

Another, and the newest feature, of the hospital is the recently opened electrical department. A

tablet on the wall of the principal room bears the following explanatory inscription: "This Electrical Department was presented to the Royal Devon and Exeter Hospital by Marianne Sanders, in memory of her husband and son, Presidents of the Hospital—19th July, 1907." Four rooms are devoted to the department, and among the instruments which are in use at present are a high frequency apparatus, a one-lamp Finsen apparatus, and an $x$-ray apparatus. There are, in addition, conveniences for giving radiant and electric baths. The department is necessarily as yet in its infancy, and few cases have so far been treated, but it is undoubtedly an important and useful adjunct to the means at the disposal of the staff for the treatment of patients.

